# Transient Receptor Potential Canonical Channels in Health and Disease: A 2020 Update

**DOI:** 10.3390/cells10030496

**Published:** 2021-02-25

**Authors:** Priya R. Kirtley, Gagandeep S. Sooch, Fletcher A. White, Alexander G. Obukhov

**Affiliations:** 1 The Department of Anatomy, Cell Biology & Physiology, Indiana University School of Medicine, Indianapolis, IN 46202, USA; prrkirtl@iu.edu (P.R.K.); gagansooch111@gmail.com (G.S.S.); 2 The Department of Anesthesia, Indiana University School of Medicine, Indianapolis, IN 46202, USA; fawhite@iu.edu; 3 Stark Neurosciences Research Institute, Indiana University School of Medicine, Indianapolis, IN 46202, USA

This 2020 Special Issue “TRPC channels” of *Cells* was dedicated to commemorating the 25th anniversary of discovery of the Transient Receptor Potential Canonical (TRPC) channel subfamily. TRPCs are calcium permeable cation channels that are localized to the plasma membrane. Cloning the first TRPC channel (TRPC1) in 1995 [1,2] opened a new vast horizon for research efforts. Since then, the accumulated knowledge has been important to understanding the roles of TRPCs in different tissues and organs of our body. In this Editorial, we briefly summarize and compare the views on TRPC channel roles in health and disease presented by the Special Issue authors.

The first hot question that attracted the attention of several authors [3,4,5,6,7,8] concerned the mechanisms of the TRPC channels’ activation. From the first days of TRPC channel discovery, two major mechanisms were initially proposed to describe the gating of TRPCs. One mechanism involved the G-protein-coupled receptors that signal in a phospholipase C (PLC)-dependent manner. It was indeed demonstrated that many TRPCs can be directly activated by diacylglycerol [9,10], a product of PLC. According to the second proposed mechanism, TRPCs are stimulated after the intracellular calcium store depletion by the store-operated calcium entry (SOCE) mechanism. Later, several other possible mechanisms were proposed, such as those involving receptor tyrosine kinases, sphingolipids, lysophospholipids, hypoosmotic shock, and mechanical stimuli. Chen et al. [11] discussed and summarized the different points of view on all those proposed signaling mechanisms. However, despite 25 years having elapsed, no consensus has been reached so far to identify the most prevalent mechanism for TRPC activation. 

Once activated, TRPCs enable monovalent cations and calcium to enter the cytosol, resulting in cell depolarization and activation of various calcium triggered proteins including kinases and phosphatases. This TRPC ability can prove to have significant effects on many tissues and organs, such as the skeletal and smooth muscle, heart, brain, kidneys, lungs, gut, and pancreas; this may contribute to pathogenesis in some cases when the channels are mutated or overactivated by some other agents like sphingolipids or lysophospholipids. A review article by Dr. Islam discussed the role of TRPC channels in the islets of Langerhans [12]. Pancreatic β-cells in the islet of Langerhans secrete insulin in a calcium-dependent manner when stimulated by glucose and incretin hormones. SOCE is suggested to play a key role in modulating insulin secretion in β-cells. Dr. Islam emphasized that TRPC1 appears to act as a SOCE channel and is the only member of the TRPC family found at the mRNA level in human pancreatic β-cells. Since SOCE is impaired in the β-cells of type 2 diabetics, it is not surprising that genetic TRPC1 polymorphisms are associated with type 2 diabetes. Additionally, Dr. Islam noted that besides TRPC1, TRPC3, and TRPC4 channels are found in mouse and rat pancreatic β-cells. TRPC3 can be triggered by stimulation of a G-protein coupled receptor GPR40, a free fatty acid receptor 1. Conversely, TRPC4 is seemingly activated by store depletion in β-cells. Activation of these channels may cause plasma membrane depolarization, enhancing glucose-stimulated membrane potential changes and calcium oscillations in β-cells. Thus, the pancreas is one of many examples in which TRPC channels can play a role in end-organ function.

In addition to modulating glucose-induced insulin secretion in the pancreas, DAG-mediated TRPC3 activation is also shown to increase insulin-mediated glucose transport in skeletal muscle t-tubules where TRPC3 is colocalized with the insulin-sensitive glucose transporter 4 [6]. A review by Choi et al. discusses this and the roles of various TRPCs in skeletal muscles [6]. It is long recognized that calcium is needed for the intricate coordination of contractile proteins during skeletal muscle contraction. Direct coupling of activated plasma membrane dihydropyridine-sensitive voltage-gated calcium channel and the ryanodine receptor (RyR) in the sarcoplasmic reticulum (SR) is critical to stimulate the calcium release from the SR, which initiates the skeletal muscle contractions. Additionally, another possible source of calcium influx in skeletal muscles is SOCE, which is induced by depletion of calcium stores leading to extracellular calcium entry that is independent of the dihydropyridine receptor. Choi at al. suggested that in muscle cells, these SOCE mechanisms can be mediated by either the Orai channels or the TRPC channels. Orai channels are store-operated channels which are highly selective to calcium and use stromal interaction molecule (STIM1) as a sensor of store depletion. Orai channels are shown to be important for skeletal muscle development and terminal differentiation through a SOCE-mediated mechanism. Orai channels are described as the “main players” of STIM-dependent SOCE. However, Choi at al. consider TRPC channels to be the “emerging players” because they have recently been found to play many roles in the formation of skeletal muscle cells. For example, TRPC3 activation in skeletal fiber type I and II was reported to regulate myoblast differentiation, while TRPC1 and TRPC4 may modulate the migration and lateral alignment of myoblasts, and myotubule formation by myoblast fusion. Moreover, a functional interaction between TRPC3 and RyR1 was reported; this may regulate the SR calcium release independent of SR calcium load. However, neither Orai nor TRPC activation is shown to provide sufficient calcium influx to activate skeletal muscle contractions. Thus, there is still much to be understood about TRPCs’ role in skeletal muscles. For example, even though TRPC6 is expressed in muscle cells, its function in skeletal muscle is yet to be determined.

The possible mechanisms of the interaction between Orai1, TRPC1, and STIM1 have been described in the review by Lopez et al. [8]. STIM1 has the STIM1-Orai1 activation region (SOAR), which is thought to interact with both TRPC1 and Orai1. SOAR of STIM1 interacts with the C-terminal and N-terminal binding sites of Orai1 when intracellular calcium stores are depleted. This allows the formation of the STIM1-Orai1 complex, which mediates calcium entry [13]. The consequential calcium influx mediated by the STIM1-Orai1 complex is proposed to promote TRPC1 translocation to the plasma membrane near the STIM1-Orai1 complex. Once on the plasma membrane, TRPC1 may be stimulated by STIM1, leading to the formation of a TRPC1-STIM1-Orai1 complex which can mediate cation non-selective Isoc currents. The STIM1-Orai1-TRPC1 complex formation is proposed to require electrostatic interaction between the negatively charged aspartate-rich region on TRPC1 and the positively charged lysine region on STIM1. Although published evidence suggests, it remains unclear whether STIM1 may directly cause TRPC1 activation. Fascinatedly, although TRPC1 and Orai1 both allegedly use STIM1 in mediating SOCE, this is not the only way TRPC and Orai channels are thought to interact. It is also suggested that TRPC channels can indirectly modulate Orai activity. This is because TRPC1 and TRPC4 are permeable to calcium and sodium, which means that the opening of these TRPC channels can lead to an influx of these both cations that would affect the electrochemical gradient. Thus, TRPC activation leads to depolarization of the plasma membrane, which subsequently decreases the driving force needed for calcium entry via Orai channels. Another mechanism of indirect modulation involves a calcium-induced inhibition of Orai channels. Thus, these theories suggest a complex and finely nuanced relationship between Orai and TRPC channels; more research into this subject is needed to clarify their roles of such interactions in various pathologies.

TRPC1 has emerged as a hotspot for upcoming research, as it also seems to be involved in regulating hippocampal neurons [3,14], cardiac cell function [5,15], and contributes to cancer progression [13]. There is a long-lasting debate concerning whether TRPC1 can be considered an ion channel by itself or simply a modulatory subunit for other TRPC channels. For example, within the hippocampus, TRPC1 channels do not normally form functional homomers in the plasma membrane [3]. Instead, they are likely to form complexes with TRPC4 and TRPC5 [14]. TRPC4 and TRPC5 are activated in a PLC-dependent pathway. Compared to TRPC4 and TRPC5 homodimers, heterodimers with TRPC1 are associated with a different shape in current–voltage relationship and a decreased permeability to calcium [3]. Recent experimental data suggested that TRPC1 and TRPC4 activation promoted neurotransmitter release at glutamatergic synapses in a calcium-dependent manner [16]. TRPC1 and TRPC5 are thought to be involved in regulating neurite outgrowth and morphology [17,18]. To determine the role of TRPC1 with and without its heterodimeric counterparts TRPC4 and TRPC5 in the neurons of the CA1 region of the hippocampus, Kepura et al. knocked out TRPC1 in these cells, while Arboit et al. knocked out TRPC4/TRPC5 [3,14]. Taken together, these two different experiments provide a cohesive picture of TRPC1 involvement in the hippocampus as outlined below.

Kepura et al. observed that deletion of TRPC1 in hippocampal CA1 neurons resulted in increased inward currents. Thus, TRPC1 has an inhibitory effect on receptor-operated non-selective cation channels. However, deletion of TRPC1 channels had a minor effect on dendritic architecture. It is thought that this inhibitory action is due to TRPC1 channel tendency to form heterodimers with other TRPC isoforms, or perhaps even that TRPC influences other endogenous non-TRPC cation channels. On the other hand, when Arboit et al. knocked out TRPC4 and TRPC5 in hippocampal CA1 pyramidal neurons, they observed that this resulted in inhibition of persistent firing. This conclusion was supported using pharmacological tools, such as TRPC4 blocker ML204, TRPC5 blocker clemizole hydrochloride, and TRPC4 and 5 blocker Pico145, which inhibited firing, as well as TRPC4 and TRPC5 antibodies, which significantly decreased persistent firing. Interestingly, although knockout of TRPC1 or TRPC4/TRPC5 is proven to have effect on hippocampal neurons, the knockout of all three TRPC channels did not change persistent firing in the medial entorhinal cortex, as found by Egorov et al. [19]. Thus, further studies are needed to investigate the role of TRPC1 in the medial entorhinal cortex.

TRPC1 is proposed to be involved in the SOCE pathway in different cardiac cells. A research paper by Bartoli et al. [5] provided evidence that the mineralocorticoid receptor (MR) modulates the expression of specific TRPC channels in adult rat ventricular cardiomyocytes. They found that after a 24-hour aldosterone treatment of rat ventricular cardiomyocytes, calcium depletion resulted in an augmented calcium entry and the upregulation of TRPC1, TRPC5, and STIM1 expression at the mRNA and protein levels. In contrast, expressions of Orai1 and Orai3 were not upregulated under the same conditions. These findings led the researchers to conclude that in adult rat ventricular cardiomyocytes, Orai1 and Orai3 expression is independent of MR, whereas the expression of TRPC1 and TRPC5 is strongly upregulated in a MR-dependent manner, supporting a previous similar finding by Hu et al. [20] who provided evidence that the MR activation increases TRPC1 and TRPC5 expression in the pig adrenal gland. 

A review by Baudel et al. [7] examined the putative role of Orai and TRPC channels in mediating SOCE in vascular smooth muscle cells. The authors concluded that SOCE in native contractile vascular smooth muscles cells is most likely mediated by TRPC1 activation with a lesser contribution of Orai channels. Specifically, the authors pointed to the role of the TRPC1/TRPC5 heterodimer formation in vascular smooth muscle cells, with TRPC1 conferring activation by store-depletion to the resulting heteromeric channel. Baudel et al. also emphasized that phosphorylation of TRPC1 by PKC and phosphatidylinositol 4,5-bisphosphate (PIP2) are obligatory for TRPC1 activity in contractile vascular smooth muscle cells. When calcium stores are depleted, STIM1 couples the Gq-PLCβ1 pathway to drive PKC-dependent phosphorylation of TRPC1 [21]. Both these articles suggested that in these specific contexts, TRPC channels, especially TRPC1, may be more prevalent in the SOCE pathway, while Orai1 seems to be less involved. 

However, while TRPC1 seems to play an important role in SOCE, Orai channels cannot be dismissed from playing a role in other cardiac cells. Camacho Londoño et al. [15] studied cardiac fibroblasts and demonstrated the importance of Orai channels over TRPC channels. Intact cardiac fibroblasts endogenously express many TRPC channels, especially TRPC1, TRPC3, and TRPC4 channels. To test their roles, Camacho Londoño et al. knocked out all seven TRPC channels and assessed angiotensin II (angII)-induced changes. Compared to control cardiac fibroblasts, the hepta-knockout TRPC cardiac fibroblasts revealed no change in calcium levels upon angII stimulation, suggesting a prevalent role of Orai channels. These findings suggest that TRPC channels may be dispensable in angII-evoked SOCE pathways in isolated cardiac fibroblasts: a stark difference to their role in the other cells discussed above. However, Camacho Londoño et al. [15] acknowledged that globally knocking out TRPC proteins too early in the development may induce other compensatory mechanisms to make up for the lack of TRPC channels. This may explain the differences in results. 

Finally, TRPC1 was demonstrated to be involved in cancer progression. Tumors often feature upregulation of calcium regulatory pathways, as an excess of calcium entry can lead to tumor proliferation, migration, metastasis, and apoptosis. Elzamzamy et al. [13] succinctly summarized that although TRPC1 may not probably represent a driver of cancer, the expression of TRPC1 may serve a hallmark of cancer. Indeed, TRPC1 is associated with cancers of the pancreas, breast, colon, lungs, and brain. However, the involvement of TRPC1 in SOCE pathways can differ based on tissue, which is a particular point of interest for the treatment of cancer. One known link of TRPC1 to cancer progression is that TRPC1 is a downstream effector of the TGFβ signaling. TGFβ plays a role in modulating a key step in tumor invasion and metastasis, epithelial–mesenchymal transition (EMT). Often, EMT is associated with upregulation of the SOCE pathway, which may involve TRPC1. This may be prevalent in pancreatic cancer cells. Although TRPC1, TRPC4, and TRPC6 were found to be upregulated in pancreatic cancer cells, only knockdown of TRPC1 led to an inhibition of pancreatic cancer cell motility [22]. Conversely, knockdown of TRPC4 and TRPC6 had no effect. However, it should be noted that EMT can also be associated with the downregulation of SOCE. This is seen in breast cancer cell lines where Orai1 is downregulated, leading to a reduction of SOCE. This reduction of SOCE was associated with EMT [23]. Notably, in this case, TRPC1 levels were unchanged. These differing results indicate that TRPC1 expression and SOCE pathways leading to cancer progression may be condition- and tissue-specific, and further research is needed to investigate what underlie such differences. 

However, when all seven TRPC channels were knocked out in the Hepta-TRPC-knockout model, a functional SOCE system still existed [24]. Thus, not only are SOCE pathways tissue-specific, but they may also be TRPC channel independent. While everyone acknowledges that perhaps TRPC1 function requires special activation mechanisms or is sometimes limited to act as a modulator, these findings open a world of questions for future experiments: when, where, and how is TRPC1 activated and what determines its involvement in SOCE pathways during cancer progression. 

Apoptosis is one of the pathways which is induced after the ischemia-reperfusion injury. One way to signal apoptosis is phosphatidylserine externalization. A plasma membrane protein called phospholipid scramblase 1 (PLSCR1) is crucial for this step. A study by Guo et al. [25] uncovered that there may be an interaction between TRPC5 and PLSCR1. Specifically, Guo et al. proposed that PLSCR1 interacts with TRPC5 through its C-terminal domain in HEK293 cells and mouse cerebrocortical neurons. Such interaction creates a PLSCR1–TRPC5 complex, which appears to promote apoptosis. This hypothesis is supported by the finding that genetic deletion of TRPC5 provided a “protective effect” during ischemia-reperfusion injury, with TRPC5 KO mice exhibiting reduced phosphatidylserine externalization and neuronal apoptotic cell death after cerebral ischemia-reperfusion injury. 

The roles of TRPC channels in dysregulation and calcium mishandling during ischemic heart disease were discussed in a review by Falcón et al. [26]. The authors noted that TRPC activation appears to play a minor role in the healthy heart. However, the expression of TRPC channels is upregulated following myocardial infarction, and their activation can contribute to subsequent fibrosis and cardiac hypertrophy responses. While Falcón et al. pointed out that TRPC6 can have “wound healing” properties and TRPC5 may be involved in angiogenesis and revascularization after ischemic events, TRPC channels do not seem to play an essential role in cardiac contractility, instead acting as regulators. 

In addition to ischemic heart disease, TRPC channels are found to have roles in classic cardiac hypertrophy, structural cardiac remodeling, fibrosis, and atrophy, as reviewed by Numanga-Tomita et al. [7]. Moreover, it is thought that TRPC channels can lead to various outcomes based on environmental stress. For instance, it is proposed that TRPC3 may specifically couple to Nox2, inducing abnormal accumulation of ROS and ultimately creating cardiac fibrosis and cardiac atrophy. Knockout of TRPC3 or Nox2 only affects cardiac fibrosis, but angII-induced cardiac hypertrophy is attenuated by the suppression of TRPC3 and Nox2. This array of effects from TRPC3 have led researchers to believe that universal signals such as ROS and calcium are not linked to one specific outcome, but rather depend on the biological context. In addition, Numanga-Tomita et al. note that pathological cardiac hypertrophy may be associated with a calcium-dependent transcriptional pathway of calcineurin/nuclear factor of activated T cells (NFAT). TRPC channels can play a role in calcineurin/NFAT signaling due to their ability to mediate calcium influx. Recent experiments suggest that TRPC4 channel isoforms have different functions in cardiomyocytes and affect downstream targets of universal calcineurin/NFAT signaling in cardiac hypertrophy, as does the upregulation of TRPC1. Additionally, TRPC3 overexpression in cardiomyocyte-specific transgenic mice exhibited elevated SOCE, NFAT activation, and cardiomyopathy.

Fascinated with the involvement of TRPC3 in the NFAT pathway, Graziani et al. [27] utilized photopharmacology and a novel photochromic TRPC benzimidazole activator (OptoBI-1) to precisely manipulate NFAT1 while activating TRPC3 in HEK293 cells. The authors reported that coupling of TRPC3 to NFAT1 activity requires global rather than local calcium changes. This is mechanistically different from the STIM/Orai pathway, which appears to function in more local manner while coupling to calcineurin/NFAT signaling. Such differences in these two pathways can provide further insight into their various roles in different tissues. 

For studying the specific contribution of TRPC3, it is important to have pharmacological tools that help to isolate a pure TRPC3 channel effect. Urban et al [4] reported that artemisinin can directly activate TRPC3 channels or TRPC3/TRPC6 heterodimers without significantly activating homomeric TRPC6 or TRPC7. Moreover, artemisinin was found to inhibit TRPC6. This is an advantage over another highly potent TRPC3 activator, 4n (for review see [11])**.** Another useful TRPC activator is engerlin-A, which specifically activates TRPC4 and TRPC5. Interestingly, the structures of TRPC4 and TRPC5 activator englerin-A and TRPC3 activator artemisinin are very similar. 

Englerin-A is also mentioned in the review by Kim et al. [28] discussing the structure-function relationship of TRPC4 and TRPC5. This review lists many key mutations that helped in identifying the function of TRPC4 and TRPC5. Kim et al. also indicated that many studies to date have aimed at determining the tetramerization mechanism in TRPC4 and TRPC5 channels. It was reported by multiple groups that the second and third ankyrin-repeat domains (ARD), helix–loop–helix domain, and connecting helix of TRPC4 are responsible for homo- or hetero-tetramerization. For TRPC5, it was determined that the domains are the second ARD and connecting helix. Unfortunately, the available Cryo-EM structures for these channels do not provide insight to the mechanisms of the heteromerization and tetramerization processes. Therefore, mutagenesis data remain to be confirmed using the Cryo-EM approach. The review emphasized that mutagenesis studies identified several key cysteine residues that are important for the function of TRPC4 and TRPC5. These are C549 and C554 residues in TRPC4 and C553 and C558 residues in TRPC5. Available Cryo-EM structures did resolve these cysteine residues. However, they were unable to confidently or consistently explain their functional implications. The review discusses some limitations of the available TRPC4 and TRPC5 structures. Firstly, all available thus far TRPC4 and TRPC5 structures are in the closed conformation, leaving the fine details of the selectivity filter and other pore residues in the dark for now. Another glaring limitation is that currently available Cryo-EMs are unable to analyze the organization of some flexible domains such as the far south domain (FSD) because it is truncated during the preparation steps. It is assumed that many important functional regions are present in FSD which is why a method to analyze them is imperative to fully grasp the structure/function relationships of TRPC channels.

Several TRPC channels are implicated in kidney function. Hall et al. [29] discusses the roles of TRPC6 in signaling pathways at the slit diaphragm in light of podocyte physiology. TRPC6 channel activation was associated with localized ROS production. Moreover, the review emphasizes that some mutations in the TRPC6 gene are associated with familial forms of kidney disease, such as diabetic nephropathy, immune-mediated kidney diseases, renal fibrosis, and focal segmental glomerulosclerosis (FSGS). Indeed, whole-body knockouts of TRPC6 reduced the disease process in glomerular, interstitial, and tubular kidney compartments. TRPC6 and TRPC3 have shown to be upregulated in glomerular diseases. Additionally, TRPC5 and TRPC6 have been shown to contribute to calcium entry in podocytes. Consequently, multiple studies have aimed to target TRPC for developing therapeutic drugs and treating such nephrological disorders. 

The role of natural mutations in TRPC receptors is summarized in the review by Liu et al. [30]. Phosphorylation, N-glycosylation, disulfide bond formation, ubiquitination, S-nitrosylation and S-glutathionylation are a few of the studied modifications that occur in TRPC channels. Phosphorylation is the most widely studied and is prevalent in all the TRPC channels. It is believed to both enhance and decrease channel activity depending on the specific channel. N-glycosylation is far less studied, but it has been shown to reduce TRPC3 and TRPC6 activity. Liu et al. indicated that disulfide bonds have been found to be important for TRPC4 and TRPC5 function. In the former, they are believed to reduce the whole cell current while also stabilizing the channel’s pore. In the latter, they are believed to be necessary for activation and multimerization of the channel. S-nitrosylation and S-glutathionylation have been identified in TRPC5. Liu et al. noted that the exact mechanism of S-nitrosylation is unclear but it is believed to be a negative regulator that suppresses the entry of calcium into endothelial cells. S-glutathionylation is believed to cause activation of a calmodulin-dependent protein kinase and calpain-caspase pathways which ultimately lead to adverse effects in the neurons. Ubiquitination of TRPC4 has been observed. However, it does not cause degradation as expected. It simply causes internalization of the channel from the plasma membrane to the intracellular compartment. Liu et al. indicated that most mutations of TRPC channels are correlated with cellular dysfunction. However, at least one missense TRPC4 SNP can supposedly confer resistance to MI in diabetic patients. 

As such, research on TRPC channels has clearly significantly expanded since the initial cloning of TRPC1 twenty-five years ago. Many important discoveries have been done in the TRPC field, allowing scientists to understand another vital molecular facet of health and disease. 
